# Medical College of the University of Buffalo

**Published:** 1846-12

**Authors:** 


*Medical College of the University of Buffalo.*—The Council of University of Buffalo have leased for a term of years the edifice on the corner of Seneca and Washington streets, for the use of the Medical Department. The proper alterations are being commenced, and will be completed in season for the first lecture term, which commences on the last Wednesday in February. The council have been fortunate in obtaining a building admirably adapted for a Medical College. Had it been erected with a view to this purpose it could hardly have been improved. A pictorial representation of the College accompanies the Annual Circular, which has lately been issued.

